# The effectiveness and cost-effectiveness of screening for latent tuberculosis among migrants in the EU/EEA: a systematic review

**DOI:** 10.2807/1560-7917.ES.2018.23.14.17-00543

**Published:** 2018-04-05

**Authors:** Christina Greenaway, Manish Pareek, Claire-Nour Abou Chakra, Moneeza Walji, Iuliia Makarenko, Balqis Alabdulkarim, Catherine Hogan, Ted McConnell, Brittany Scarfo, Robin Christensen, Anh Tran, Nick Rowbotham, Marieke J van der Werf, Teymur Noori, Kevin Pottie, Alberto Matteelli, Dominik Zenner, Rachael L. Morton

**Affiliations:** 1 Division of Infectious Diseases, Jewish General Hospital, McGill University, Montreal, Canada; 2 Centre for Clinical Epidemiology of the Lady Davis Institute for Medical Research, Jewish General Hospital, Montreal, Canada; 3 Department of Infection, Immunity and Inflammation, University of Leicester, Leicester, United Kingdom; 4 Division of Infectious Diseases, Sherbrooke, Canada; 5 Musculoskeletal Statistics Unit, The Parker Institute, Bispebjerg and Frederiksberg Hospital, Copenhagen, Denmark; 6 Department of Rheumatology, Odense University Hospital, Denmark; 7 National Health and Medical Research Council, NHMRC Clinical Trials Centre, University of Sydney, Sydney, Australia; 8 European Centre for Disease Prevention and Control, Stockholm, Sweden; 9 Bruyere Research Institute, University of Ottawa, Ottawa, Ontario, Canada; 10 Clinic of Infectious and Tropical Diseases, University of Brescia and Brescia Spedali Civili General Hospital, World Health Organization Collaborating Centre for TB/HIV and TB Elimination, Brescia, Italy; 11 Respiratory Diseases Department, Centre for Infectious Disease Surveillance and Control (CIDSC), Public Health England, London, United Kingdom; 12 Department of Infection and Population Health, University College London, London, United Kingdom

**Keywords:** latent TB, migrant, screening, EU/EEA, tuberculosis

## Abstract

Migrants account for a large and growing proportion of tuberculosis (TB) cases in low-incidence countries in the European Union/European Economic Area (EU/EEA) which are primarily due to reactivation of latent TB infection (LTBI). Addressing LTBI among migrants will be critical to achieve TB elimination. **Methods:** We conducted a systematic review to determine effectiveness (performance of diagnostic tests, efficacy of treatment, uptake and completion of screening and treatment) and a second systematic review on cost-effectiveness of LTBI screening programmes for migrants living in the EU/EEA. **Results:** We identified seven systematic reviews and 16 individual studies that addressed our aims. Tuberculin skin tests and interferon gamma release assays had high sensitivity (79%) but when positive, both tests poorly predicted the development of active TB (incidence rate ratio: 2.07 and 2.40, respectively). Different LTBI treatment regimens had low to moderate efficacy but were equivalent in preventing active TB. Rifampicin-based regimens may be preferred because of lower hepatotoxicity (risk ratio = 0.15) and higher completion rates (82% vs 69%) compared with isoniazid. Only 14.3% of migrants eligible for screening completed treatment because of losses along all steps of the LTBI care cascade. Limited economic analyses suggest that the most cost-effective approach may be targeting young migrants from high TB incidence countries. **Discussion:** The effectiveness of LTBI programmes is limited by the large pool of migrants with LTBI, poorly predictive tests, long treatments and a weak care cascade. Targeted LTBI programmes that ensure high screening uptake and treatment completion will have greatest individual and public health benefit.

## Introduction

Tuberculosis (TB) control programmes in the European Union/European Economic area (EU/EEA) have successfully managed to reduce TB rates by 50% over the past 20 years [1-4]. Although EU/EEA countries are committed to the ambitious World Health Organisation (WHO) goal of TB elimination, the rate of TB decline of 4.3% per year over the past decade (2007–2016) in the region is insufficient to achieve this goal [1-5]. It is projected that a mean decline of 18% per year will be necessary to meet the WHO goal and that TB control strategies must be scaled up, including addressing the burden of latent TB infection (LTBI) [3,5,6].

The foreign-born population makes up an increasing and considerable number and proportion of all TB cases in EU/EEA countries with a low TB incidence (< 10 cases/100,000 population) [7]. The majority of these cases are due to reactivation of LTBI acquired in the patients’ countries of origin. Although foreign-born people make up 11.4% of the population in the EU/EEA, they represented more than one quarter of reported TB cases in 2015 [4,8,9]. This burden is even greater in EU/EEA countries with low TB incidence where often more than half of all reported TB cases occur in migrants [4]. This is because a considerable proportion of migrants were born in high TB burden countries where 26–46% of the population are latently infected with TB [4,10-13]. The WHO has only conditionally recommended LTBI screening among migrants living in low TB burden countries (< 100 cases/100,000 population) owing to reservations about implementation and the low quality of evidence of the effectiveness and cost-effectiveness of LTBI programmes in these settings [6]. Screening the potentially large pool of latently infected migrants and treating those found to be positive poses an enormous challenge in the EU/EEA, especially since less than half of these countries have such programmes [11,14,15]. The aim of this study was to conduct a systematic review on the effectiveness and cost-effectiveness of screening for latent TB among migrants to the EU/EEA to inform migrant screening guidelines.

## Methods

### Overall approach and key questions

**Figure 1:**
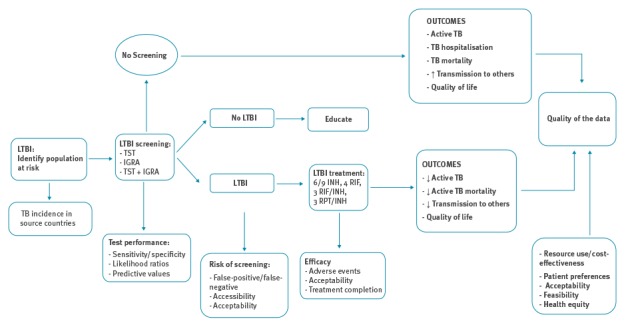
Analytic framework for latent tuberculosis screening in migrants

IGRA: interferon gamma release assay; INH: isoniazid; LTBI: latent tuberculosis infection; RIF: rifampicin; RPT: rifapentine; TB: tuberculosis; TST: tuberculin skin test.

This review supports a project of the European Centre for Disease Prevention and Control (ECDC) to develop guidance on screening for six infectious diseases (chronic hepatitis C, hepatitis B, HIV, TB (active and latent), and intestinal parasites) in newly arrived migrants to the EU/EEA. The project followed the new Grading of Recommendations Assessment, Development and Evaluation (GRADE)-ADOLOPMENT approach to conduct systematic reviews on screening migrant populations for these six infectious diseases [16]. The review protocol and the methods of ADOLOPMENT guideline development have been published [16,17]. All reviews followed a Cochrane methodological approach and the Preferred Reporting Items for Systematic Reviews and Meta-Analyses (PRISMA) methods for reporting systematic reviews [18]. For this review, we developed research questions (PICO), an analytic framework to illustrate the screening evidence pathway, and identified and prioritised clinically-important outcomes [19]. These evidence-based review methods were first described by the United States (US) Preventative Task Force [19,20]. We sought to answer two research questions: (i) what is the effectiveness of screening migrants arriving or living in the EU/EEA for LTBI and (ii) what is the resource use, costs and cost-effectiveness of screening migrants for LTBI? To address these questions, we developed an analytic framework (Figure 1) and the following key questions along the LTBI screening evidence pathway: (i) what are the test properties of LTBI screening tests: tuberculin skin test (TST), interferon gamma release assay (IGRA) or sequential TST/IGRA, (ii) what are the efficacy and harms of LTBI therapies, (iii) what is the uptake of screening and treatment and completion of treatment, and (iv) what is the cost-effectiveness of LTBI screening and treatment for migrants [17].

### Search strategy and selection criteria

Following the GRADE-ADOLOPMENT process, we identified an evidence review that assessed the effectiveness of latent TB infection (LTBI) screening among migrants, published in 2011 by the Canadian Collaboration on Immigrant and Refugee Health (CCIRH), and used this as a starting point for our literature search (anchoring review) [16,21]. The CCIRH review included systematic reviews on the effectiveness of LTBI screening in migrants up to 2008 but did not review cost-effectiveness. We therefore conducted two separate searches to address our research questions. The first search updated the CCIRH evidence review and identified systematic reviews and guidelines on the effectiveness and cost-effectiveness of TB screening programmes in migrant populations from 2005 to 2016. The second search identified individual studies on the resource use, costs and cost-effectiveness of TB screening programmes for migrants over a longer time, 2000 to 2016, given these topics were not covered in the CCIRH evidence review. For the first search, MEDLINE via Ovid, Embase, the Cumulative Index to Nursing and Allied Health Literature (CINAHL), Epistemonikis, and Cochrane CENTRAL between 1 January 2005 and 12 May 2016 were searched for evidence on the effectiveness and cost-effectiveness of LTBI screening programmes in migrants. We used a combination of key terms including: ‘tuberculosis’, ‘screening’, ‘chest-radiograph’, ‘tuberculin skin test’, ‘interferon-gamma release assays’, ‘costs’, ‘cost-effectiveness’ AND ‘guidelines’, ‘reviews’. The search terms and strategy in Ovid MEDLINE are included in Supplement 1. We also searched grey literature and published guidelines and reports at the US Centres for Disease Control and Prevention (CDC), ECDC, WHO, and the International Union Against Tuberculosis and Lung Disease (IUATLD). We did not apply language restrictions to the search. Additional guidelines and studies were identified by our co-authors and through searching bibliographies of included studies. In the second search, using the search terms on ‘tuberculosis’, ‘screening’, ‘costs’ and ‘cost-effectiveness’, we searched MEDLINE, Embase, the National Health Service Economic Evaluation Database (NHS EED), the Database of Abstracts of Reviews of Effects (DARE) and the Tufts Medical Center Cost Effectiveness Analysis Registry and Google scholar databases between 1 January 2000 and 31 May 2016.

### Study selection and quality assessment

We identified and included systematic reviews and evidence-based guidelines that directly addressed each key question along the LTBI screening evidence chain (Figure 1) and prioritised those focusing on newly arrived (< 5 years in the host country) migrants. Migrant populations included non-forced economic migrants, refugees and asylum seekers, and illegal migrants who may have been forced to flee conflict, natural disaster, or economic peril [17]. We only included studies published in full and in English or French. If more than one version of a systematic review was identified, the most recent was considered. Studies were excluded if there were not relevant to the key questions, if they were not a systematic review or guideline, if the study methodology was unclear, and if they focussed only on non-generalisable subgroups (such as healthcare workers or HIV-positive people) or addressed only active TB screening. Two authors screened the titles and abstracts, assessed selected full-text articles for eligibility and extracted data from included articles. Disagreements were resolved by consensus or by a third author. The methodological quality of systematic reviews was assessed using the AMSTAR tool (A Measurement Tool To Assess Systematic Reviews) and the quality of individual studies was assessed with the Newcastle-Ottawa scale [22,23]. The GRADE criteria were applied to assess the quality and certainty of the evidence of the individual studies included in the systematic reviews [24].

### Data extraction and synthesis

The following information was extracted from each study; study design, objectives, analyses, quality of the individual studies included in the systematic review, population examined, number of included studies, total number of participants included, intervention, outcome and results. We created GRADE evidence profiles and summary of findings tables for each outcome where appropriate.

For each of the cost-effectiveness studies we extracted the following data: economic methods used (e.g. micro-costing study, within-trial cost-utility analysis, Markov model), description of the case base population, the intervention and the comparator, absolute size and relative difference in resource use, and cost-effectiveness results (e.g. incremental net benefits (INB) or incremental cost-effectiveness ratio (ICER)) [25]. The certainty of economic evidence in each study was assessed using the relevant items from the 1997 Drummond checklist [26]. All currencies were converted to 2015 Euros using the Cochrane web-based currency conversion tool: https://eppi.ioe.ac.uk/costconversion/default.aspx.

## Results

### Search results

**Figure 2:**
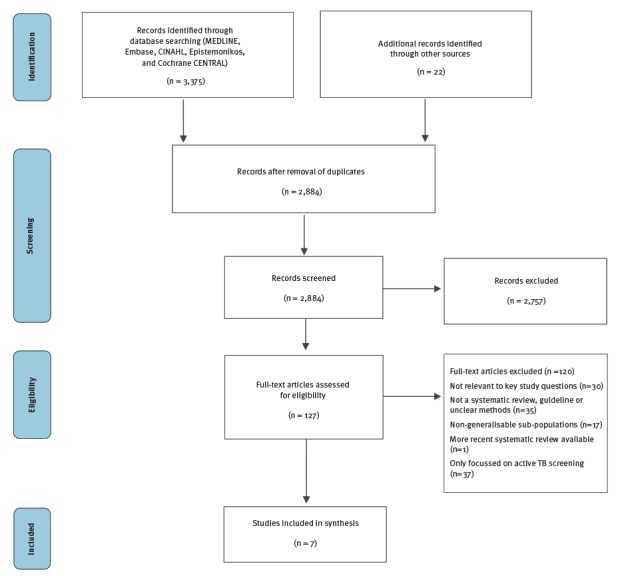
PRISMA flow diagram, literature search for the effectiveness and cost-effectiveness of latent tuberculosis screening, 1 January 2005–12 May 2016

CINAHL: Cumulative Index to Nursing and Allied Health Literature; TB: tuberculosis.

**Figure 3:**
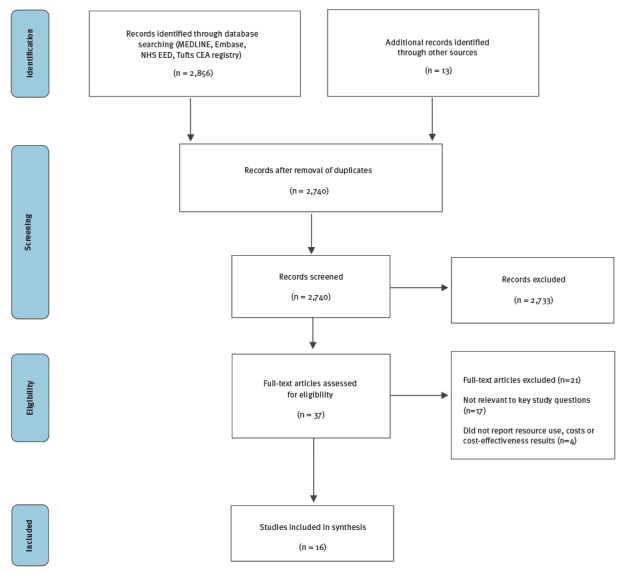
PRISMA flow diagram, literature search for the resource use, costs and cost-effectiveness of latent tuberculosis, 1 January 2000–31 May 2016

In the first search on the effectiveness and cost-effectiveness of TB screening programmes in migrants, we retrieved 3,375 studies and identified 22 additional records through other sources on the effectiveness of latent TB screening in migrant populations (Figure 2). After removal of duplicates, 2,884 studies were screened by title and abstract. A total of 127 studies were selected for full text assessment. We did not identify any single study on the effectiveness of LTBI screening in migrants or the general population. We therefore included seven systematic reviews that addressed the LTBI screening chain of evidence; the test properties of LTBI screening tests (n = 3) [20,27,28], the efficacy and harms of LTBI therapies (n = 2) [29,30], and the LTBI care cascade including uptake of screening and treatment initiation and completion (n = 2) [31,32]. In the economic search 2,869 articles were identified. After duplicate removal 2,740 articles were screened by title and abstract (Figure 3). A total of 37 studies underwent full text assessment and 16 individual studies were included [33-48].

NHS EED: National Health Service Economic Evaluation Database (NHS EED), Tufts CEA: Tufts Medical Centre Cost-Effectiveness Analysis Registry.

### Performance of diagnostic tests for latent tuberculosis infection

Three systematic reviews assessed the properties of the diagnostic tests used in LTBI screening (Table 1). The systematic reviews by Pai et al. and Kahwati et al. evaluated the performance of TST and IGRA in populations not vaccinated with bacillus Calmette–Guérin (BCG) and found that the TST, at a 10 mm cut-off, and IGRA had similar and good sensitivity (79%) and high specificity (> 97%) to detect LTBI [20,27]. In addition, Pai et al. showed that the TST was limited by lower specificity (59%) in BCG-vaccinated populations [27]. The third systematic review by Kik et al. estimated the ability of TST or IGRA to predict the risk of developing active TB among those with LTBI [28]. We included and present the data from eight of the 29 studies in the Kik review as they were the only ones that performed both TST and IGRA in the same study subjects and compared the results to those with a negative test [28]. The positive predictive value (PPV) and the pooled incidence rate ratios (IRR) estimated by comparing test-positive and -negative cohorts were similar for TST and IGRA. Both predicted the development of active TB poorly [28]. The PPV (range) and the IRR (95% CI) were, respectively, 1–7% and 2.07 (1.38–3.11) for the TST and 0–13% and 2.40 (1.26–4.60) for the IGRA [28].

**Table 1:** Characteristics of included studies for the effectiveness of latent tuberculosis screening, 2005–2016

Study	Quality/certainty of evidence	Design	Population	Intervention/outcomes	Results
Kahwati et al. 2016 [20]	Quality of systematic review AMSTAR: 6/11. Quality of data of included individual studies: fair to good as assessed by predefined criteria developed by USPSTF.	Systematic review up to 2016. Number of studies: n = 50 on sensitivity, n = 18 on specificity.	Asymptomatic adults at increased risk for active TB: Sensitivity n = 4,167 Specificity n = 10,693	Intervention: TST (5 mm, 10 mm, 15 mm), IGRA (T-SPOT.TB, QFT-2G, QFT-3G). Outcomes: Sensitivity, specificity (95% CI).	Sensitivity, specificity (95% CI) of LTBI screening tests: TST (5 mm): sensitivity: 79% (69–89), specificity 30–97%; TST (10 mm): sensitivity: 79% (71–87), specificity: 97% (96–99); TST (15 mm): sensitivity: 52% (35–68), specificity: 99% (98–99); IGRA (T-SPOT.TB): sensitivity: 90% (87–93), specificity: 95% (92–98); IGRA (QFT-2G): sensitivity: 77% (74–81), specificity: 98% (90–1.0); IGRA (QFT-3G): sensitivity: 80% (77–84), specificity 97% (94–99).
Pai et al. 2008 [27]	Quality of systematic review AMSTAR: 5/11. Quality of data of included individual studies: very low as assessed by GRADE.	Systematic review up to 31 March 2008, English language restriction: n = 38 studies, 3 studies QFT in high TB incidence countries.	BCG-vaccinated; Not BCG-vaccinated; n = 1,879	Intervention: TST, IGRA (QFT-2G, QFT-3G, T-SPOT.TB). Outcomes: Sensitivity, specificity (95% CI).	Sensitivity, specificity (95% CI) of LTBI screening tests: TST overall: sensitivity: 77% (71–82). TST in BCG-vaccinated: specificity: 59% (46–73). TST in non-BCG-vaccinated: specificity: 97% (95–99). IGRA (QFT): sensitivity: 76% (72–80), specificity: 98% (96–99). IGRA (QFT-2G): sensitivity: 78% (73–82). IGRA (QFT-3G): sensitivity: 70% (63–78). IGRA in BCG-vaccinated: specificity: 96% (94–98). IGRA in non-BCG-vaccinated: specificity: 99% (98–100). IGRA (T-SPOT.TB/ ELISpot): sensitivity: 90% (86–93), specificity: 93% (86–100). IGRA (T-SPOT.TB): specificity: 87% (80–92).
Kik et al. 2014 [28]	Quality of systematic review AMSTAR: 7/11. Quality of data of included individual studies: low as assessed by GRADE.	Systematic review 1999 to February 2014: n = 29 studies, 19 prospective cohorts, only 8/29 studies compared TST/IGRA head to head.	Persons at high risk of LTBI, not on tuberculosis preventive therapy: Low TB incidence^a^ < 100/100,000 High TB incidence^a^ > 100/100,000; High/intermediate incidence^a^ > 40/100,000; n = 54,833	Intervention: IGRA, TST. Outcomes: PPV, NPV, RR (number of cases in those with positive test vs those with negative test), IRR (rate of disease in those with positive test vs those with negative test).	Screening tests characteristics: The pooled RR estimate: TST: 2.64 (95%CI: 2.04–3.43), IGRA: 8.45 (95% CI: 4.13–17.3). The PPV: TST: 1–7%, IGRA: 0–13%. The NPV: TST: 92–100%, IGRA: 88–100%. The pooled IRR: TST: 2.07 (95% CI: 1.38–3.11), IGRA: 2.40 (95% CI: 1.26–4.60).
Stagg et al. 2014 [29]	Quality of systematic review AMSTAR: 8/11. Quality of data of included individual studies: unclear or high risk of bias for efficacy; evidence sparse for hepatotoxicity as assessed by Cochrane risk of bias tool.	Systematic review up to January 2014: n = 53 studies	Patients with LTBI: n patients by regimen: range: 14 (RFB-INH)–47,489 (placebo).	Interventions: INH 3–4, 6, 9, 12–74 months, RFB-INH, RPT-INH, RMP, RMP-INH 1 month, RMP-INH 3–4 months, RMP-INH-PZA, RMP-PZA, INH-EMB. Outcome: prevention of active TB; OR (95% CrI); risk of hepatotoxicity.	Various therapies containing RMP for ≥ 3 months were efficacious at preventing active TB. Regimens containing RMP may be effective alternatives to INH monotherapy. Compared with placebo, OR (95% CrI): INH 6 months: 0.64 (0.48–0.83), INH 12–72 months: 0.52 (0.41–0.66), RMP: 0.41 (0.18–0.86), RMP-INH 3–4 months: 0.52 (0.34–0.79).
Sharma et al. 2014 [30]	Quality of systematic review AMSTAR:11/11. Quality of data of included individual studies: very low to moderate as assessed by GRADE.	Systematic review up to December 2012: n = 10 studies	HIV-negative with LTBI: 10,717 patients, 2–5 years follow-up.	Interventions: RMP 3–4 months, RMP + INH 3 months vs INH 6–9 months, RMP + PZA 2 months vs INH 6 months, RFP 900 mg weekly for 3 months + INH 900 mg for 9 months. Outcome: rates of active TB/1,000, 5 years follow-up, treatment limiting adverse events, hepatotoxicity/1,000.	Effectiveness in preventing active TB, rate/1,000, RR (95% CI): RMP: 121 vs 150/1,000, RR = 0.81 (0.47–1.4); RMP + INH: 162 vs 150/1,000, RR = 1.08 (0.65–1.79); RMP + PZA vs INH: 61 vs 47/1,000, RR = 1.32 (0.42–4.13); RFP + INH: 2 vs 4/1,000, RR = 0.44 (0.18–1.07). The directly observed, shorter regimen had higher treatment completion: 82% vs 69%, RR = 1.19 (1.16 to 1.22). Hepatotoxicity: RMP vs INH, RR = 0.15 0.07–0.4).
Alsdurf et al. 2016 [31]	Quality of systematic review AMSTAR: 3/11. Quality of data of included individual studies: not reported but several gaps and limitations highlighted.	Systematic review 1946 to April 2015: Total: n = 58 studies described, 70 distinct studies: 34 prospective 36 retrospective. TST: 60 cohorts IGRA (+/− TST), 6 cohorts, testing not reported in 4 cohorts.	Patients with LTBI: 748,572 patients.	Intervention: TST, IGRA. Outcomes: number of people eligible for screening tested; number who initiated and completed screening with IGRA or TST; number with positive tests who had chest radiographic and medical evaluation; number who were prescribed, started, and, completed treatment.	Steps in the TB cascade of care associated with greater losses included: Completion of testing: 71.9%, 95% CI: 71.8–72.0; Completion of medical evaluation: 43.7%, 95% CI: 42.5–44.9; Recommendation for treatment: 35.0%, 95% CI: 33.8–36.4; Completion of treatment if started: 18.8%, 95% CI: 16.3–19.7. Steps with fewer losses included: receiving test results, referral for evaluation if test positive and accepting to start therapy if recommended. Factors associated with fewer losses included: having immunocompromising medical indications, being part of contact investigations, use of rifamycin-based regimens.
Sandgren et al. 2016 [32]	Quality of systematic review AMSTAR: 7/11. Quality of data of included individual studies: low to moderate as assessed by Cochrane risk of bias tool.	Systematic review up to February 2014, English, French, Spanish, German, and Dutch: n = 95 studies, 43 prospective, 52 retrospective. 45 studies on initiation rates, 20 were prospective. 83 studies on completion rates, 39 were prospective.	General population, case contacts, health workers, homeless, drug users, HIV-positive, inmates, immigrants, and patients with comorbidities n = not reported.	Intervention: short intervention: ≤ 4 months RMP or 2 months RMP + PZA; long intervention: (≥ 4 months) 6–9 months INH; combined intervention. Outcomes: treatment initiation rate, treatment completion rate.	Range of initiation rate and completion rate: General population: 26–99%, 39–96%; Case contacts: 40–95%, 48–82%; Healthcare workers: 47–98%, 17–79%; Homeless: 34–90%, 23–71%; Intravenous drug users: 52–91%; 38–89%; HIV-infected: 67–92%, 55–95%; Inmates: 7–90%, 4–100%; Immigrants: 23–97%, 86%; Patients with comorbidities: 82–93%, 75–92%.

AMSTAR: A MeaSurement Tool to Assess systematic Reviews [22]; BCG: Bacillus Calmette–Guérin; CI: confidence interval; CrI: credible interval; ELISpot: Enzyme-Linked ImmunoSpot; EMB: ethambutol; GRADE: The Grading of Recommendations Assessment, Development and Evaluation; HIV: human immunodeficiency virus; IGRA: interferon gamma release assay; INH: isoniazid; IRR: incidence rate ratio; LTBI: latent tuberculosis infection; NPV: negative predictive value; OR: odds ratio; PPV: positive predictive value; PZA: pyrazinamide; QFT: QuantiFERON; QFT-2G: QuantiFERON-TB Gold; QFT-3G/ QFT-GIT: QuantiFERON-TB, Gold In-Tube; RFB: rifabutin; RFP: rifampicin; RPT: rifapentine; RMP: rifampicin; RR: risk ratio; TB: tuberculosis; T-SPOT.TB: ELISPOT assay for tuberculosis; TST: tuberculin skin test; USPSTF: United States Preventive Services Task Force.

^a^ Low, intermediate and high TB incidence as defined by [28].

### Efficacy and harms of therapy for latent tuberculosis infection

Two systematic reviews examined the efficacy and associated harms of latent TB therapies to prevent the development of active TB [29,30]. Both reviews found that the efficacy of several different regimens of rifampicin (RIF) (monotherapy and combinations) was low to moderate and equivalent to isoniazid (INH) treatment for 6–12 months. Stagg et al. published a network meta-analysis of 53 randomised controlled trials on the efficacy and harms of different latent TB regimens in which 42 were directly compared [29]. In the meta-analysis of the nine placebo-controlled trials, the odds of developing active TB among those who took INH for 6 months compared with placebo were 0.64 (95% CI: 0.48–0.83). In the network meta-analysis of all 53 studies, the odds of developing active TB in the 3–4 months of RIF regimen compared with placebo were 0.41 (0.18–0.86) [29]. The Cochrane review by Sharma et al. found similar efficacy for the following three comparisons: (i) RIF monotherapy for 3–4 months vs INH for 6–9 months, (ii) RIF + INH for 3 months vs INH for 6–9 months and (iii) weekly rifapentine (RFP) + INH for 3 months vs INH for 9 months. The comparative relative risks (RR) with 95% CI for these rifamycin combinations vs INH were 0.81 (0.47 to 1.4), 1.08 (0.65 to 1.79) and 0.44 (0.18 to 1.07), respectively [30]. In that review, the RIF-based regimens were better tolerated, with lower RR of hepatotoxicity (0.15; 95% CI: 0.07–0.4), and had better adherence (82% vs 69%, RR = 1.19 (95% CI: 1.16–1.22)) [30].

### Latent tuberculosis infection care cascade: screening uptake and completion of therapy

Two systematic reviews reported on the LTBI care cascade including the uptake of screening and treatment as well as initiation and completion of therapy [31,32]. Alsdurf et al found that only 18.8% of all those eligible for screening completed LTBI therapy and that the rate was low for all sub-groups, including migrants (14.3%) [31]. This was due to progressive losses at all stages of the care cascade: 71.9% (95% CI: 71.8–72.0) completed testing, 43.7% (95% CI: 42.5–44.9) completed medical evaluation, 35.0% (95% CI: 33.8–36.4) were recommended for treatment and 18.8% (95% CI: 16.3-19.7) completed treatment if started [31]. Sandgren et al. found that treatment initiation (23–97%) and treatment completion (7–86%) varied widely among migrants [32].

### Resource use, cost and cost-effectiveness of screening for latent tuberculosis infection

The cost-effectiveness analysis of studies summarised in our review focused primarily on comparisons between LTBI screening strategies (e.g. TST, IGRA or sequential TST/IGRA), comparisons with other screening techniques such as chest radiography (CXR) for active TB, a combination of CXR/TST, or no screening, among different risk groups (Table 2). The strategies compared were heterogeneous across most studies. Eleven of the 16 included studies addressed an LTBI screening strategy and included a migrant group; however, only three studies were specifically about migrants in EU/EEA countries [35,40,48]. The cost-effectiveness of screening strategies was dependant on test characteristics, which tests were being compared, the cost of tests and whether or not the population was BCG-vaccinated.

**Table 2:** Characteristics of included studies for the resource use, costs and cost-effectiveness of latent tuberculosis screening, 2000–2016

Study	Certainty of economic evidence based on the Drummond criteria [26]	Methods /population	Intervention(s)	Cost-effectiveness (ICER or INB) per case prevented	How large are the resource requirements (costs)
Schwartzman et al. 2000 [47]	Certainty of evidence: moderate allowance was made for uncertainty in the estimates of costs and consequences and ranges were provided. No PSA were performed. Justification was provided for the range of values varied in one-way sensitivity analyses. The cost-effectiveness results were sensitive to model inputs including the probability of INH prescribed: probability of INH treatment completed, cost of inpatient treatment, TB infection rate and HIV seropositivity.	Method: decision-analytic Markov model, 20-year time horizon, 3% discount rate, perspective of the third-party payer (central and provincial governments), scenario analysis based on INH completion conducted. Population: 20-year-old immigrants to Canada originating from sub-Saharan Africa, South-east Asia, western Europe.	Three strategies: (i) No screening (ii) CXR (iii) TST	ICER (CAD/case prevented): Population 1 (50% TB-infected, 10% HIV-positive): TST vs CXR: CAD 32,601 (EUR 29,990; CXR vs no screening: CAD 3,943 (EUR 3,627). Population 2 (50% TB-infected, 1% HIV-positive): TST vs CXR: CAD 66,759 (EUR 61,413); CXR vs no screening: CAD 10,627 (EUR 9,776). Population 3 (5% TB-infected, 1% HIV-positive): TST vs CXR: CAD 68,799 (EUR 63,290); CXR vs no screening: CAD 236,496 (EUR 217,558)	Costs were large in populations 1 and 2, moderate in population 3. Costs per 1,000 patients: Population 1 (50% TB-infected, 10% HIV-positive): TST: CAD 436,390 (EUR 401,445); CXR: CAD 338,310 (EUR 311,219); No screening: CAD 332,020 (EUR 305,432). Population 2 (50% TB-infected, 1% HIV-positive): TST: CAD 342,730 (EUR 315,284); CXR: CAD 231,430 (EUR 212,897; No screening: CAD 218,250 (EU 200,773). Population 3 (5% TB-infected, 1% HIV-positive): TST: CAD 62,640 (EUR 57,623); CXR: CAD 51,170 (EUR 47,072); No screening: CAD 21,820 (EUR 20,072).
Oxlade et al. 2007 [41]	Certainty of evidence: moderate allowance was made for uncertainty in the estimates of costs and consequences; ranges were provided. No PSA was performed. One-way or two-way sensitivity analyses using higher or lower costs, other discount rates, test performance characteristics were undertaken. The cost-effectiveness results were sensitive to TST sensitivity; and risk of re-activation.	Method: decision-analytic Markov model, 20-year time horizon, 3% discount rate Canadian health system perspective, costs reported in 2004 Canadian dollars. Population: foreign-born entrants to Canada, close contacts of active TB cases.	Five strategies: (i) CXR (ii) No screening (iii) TST (iv) QFT (v) TST followed by QFT if TST-positive	CXR vs no screening: more cost-effective for screening immigrants; ICER: CAD 875/case prevented (EUR 690); QFT vs TST: cost-effective in BCG-vaccinated close-contacts and casual contacts; Sequential TST/QFT vs QFT alone is cost-effective in all scenarios; Sequential screening vs TST or QFT alone: cost-saving in screening migrants from low-incidence countries.	Low to moderate costs in immigrants from medium- and high-incidence countries. High costs in immigrants from low-incidence countries. QFT. Low incidence: CAD 64,920 (EUR 51,265); High incidence: CAD 459,040 (EUR 362,488). TST: varied based on specificity and BCG-status (and age at BCG vaccination): Non-vaccinated: CAD 30,320 (EUR 23,942); Low incidence, vaccinated older age: CAD 465,260 (EUR 367,400); Sequential TST then QFT: range from CAD 27,369 (EUR 21,612) to CAD 458,475 (EUR 362,042).
Dasgupta et al. 2000 [46]	Certainty of evidence: Low Limited allowance was made for uncertainty in the estimates of costs and consequences; ranges were provided. No PSA was performed No one-way or two-way sensitivity analyses using higher or lower costs, other discount rates, or comparisons with no screening were performed. Scenario analyses undertaken. The cost-effectiveness results were sensitive to costs for passive diagnosis of TB; Isonazid prescription rate; screening referral criteria; future risk of active TB.	Method: Cost-effectiveness analysis based on prospective non-randomised cohorts; results reported in Canadian dollars. Prospective cohort study over 1 year of costs and outcomes in 3 groups (all applicants, inactive TB requiring surveillance, and close contacts) Population: Immigration applicants undergoing CXR screening; and already arrived immigrants requiring screening for LTBI, and close contacts of active cases resident in Montreal, Quebec, Canada.	Three strategies: (i) CXR in migrants applying for a permanent residence (ii) Surveillance CXR +/− TST (iii) Close contacts CXR +/− TST	CAD/per disease prevented: Applicants: costs CAD 39,409 (EUR 36,667); Surveillance: costs CAD 65,126 (EUR 60,594); Close contacts: savings CAD 2,186 (EUR 2,033).	Applicants: moderate costs; Surveillance: large costs; Close contacts: moderate savings. Total programme costs for TB disease prevented: Applicants: CAD 73,125 (EUR 68,037); Post-landing surveillance: CAD 155,729 (EUR 144,894); Close contacts: CAD 29,668 (EUR 27,603).
Iqbal et al. 2014 [33]	Certainty of evidence: low. No allowance for uncertainty. No PSA performed. No justifications provided for ranges in cohort estimates. No sensitivity analyses for cost-effectiveness estimates.	Method: costing comparison study. Population: US- and foreign-born populations, ≥ 18-years-old with positive TST and normal CXR without TB-related symptoms.	Two strategies: (i) TST (ii) QFT	TST: less expensive in US-born patients; QFT-G: less expensive relative to TST in foreign-born individuals. No ICER or INB reported.	Moderate to large costs in US-born individuals, and large costs in foreign-born individuals. Total costs per 1,000 patients: In US-born individuals: QFT: USD 88,420 (EUR 78,200); TST: US 63,388 (EUR 56,061). In foreign-born individuals: TST: USD 313,806 (EUR 277,535); QFT: USD 177,860 (EUR 157,302).
Linas et al. 2011 [36]	Certainty of evidence: moderate allowance was made for uncertainty in the estimates of costs and consequences; ranges were provided. No PSA was performed. Limited justification for ranges used in one and two-way sensitivity analyses were provided. The cost-effectiveness results were sensitive to patient age and rates of TB reactivation, sensitivity of IGRA, IGRA test cost, adherence to INH therapy and quality of life (utility) post active TB.	Method: decision-analytic Markov model, US healthcare perspective, costs in 2011 US dollars, 3% discount rate. Population: recent immigrants (adults and children), foreign-born residents living in the US for more than 5 years, close contact adults and children, individuals with HIV, homeless, injection drug users, former prisoners, gastrectomy patients, underweight patients, individuals with silicosis, diabetes or end-stage renal disease.	Four strategies: (i) No Screening (ii) TST (iii) IGRA (iv) Screening high-risk groups	ICER (USD/QALY): Child close contacts: TST vs no screening: USD 6,200 (EUR 5,166); IGRA vs TST: USD 21,100 (EUR 17,582). Adult close contacts: TST vs no screening: USD 8,900 (EUR 7,416); IGRA vs TST: USD 21,500 (EUR 17,915). Foreign-born individuals: IGRA dominated TST; IGRA vs no screening: < USD 70,000 (EUR 58,329). Recent immigrant children and adults: IGRA dominated TST; IGRA vs no screening: Adult immigrants: US 35,200 (EUR 29,331); Children: USD 74,800 (EUR 62,328).	Total costs and resource requirements not reported.
Pareek et al. 2012 [48]	Certainty of evidence: moderate allowance was made for uncertainty in the estimates of costs and consequences; ranges were provided. No PSA was performed. Justification for ranges used in one and two-way sensitivity analyses were provided. The cost-effectiveness results were sensitive to diagnostic specificity of screening tests; proportion of immigrants commencing and completing treatment; costs of screening for LTBI.	Method: decision-analytic model, inputs derived from cohort study of immigrants in London, 20-year time horizon, costs in 2010 GB pounds. Population: migrants registered with one of four participating primary care practices in London, England between October 2008 and June 2010	Four strategies: (i) No port-of-entry CXR (ii) Port-of-entry CXR (iii) QFT (iv) T-SPOT.TB	The two most cost-effective screening strategies: No port-of-entry CXR + single-step QFT-GIT at incidence of 250/100,000: ICER of GBP 21,565/case averted (EUR 26,105); No port-of-entry CXR + single-step QFT-GIT at 150/100,000 incidence: ICER: GBP 31,867/case averted (EUR 38,576).	Moderate to large costs for the two listed (cost-effective) single-step QFT strategies. At the incidence threshold, total costs: 250/100,000: GBP 839,713 (EUR 1,016,518); 150/100,000: GBP 1,089,177 (EUR 1,318,508). Total costs per 10,000 screened: No screening: GBP 659,609 (EUR 798,493) T-SPOT.TB (+CXR at port of arrival): GBP 2,189,912 (EUR 2,651,009)
Pareek et al. 2011 [35]	Certainty of evidence: moderate allowance was made for uncertainty in the estimates of costs and consequences; ranges were provided. No PSA was performed. Justification for ranges used in one-way sensitivity analyses was provided. The cost-effectiveness results were robust to all ranges tested.	Method: decision-analytic Markov model, UK NHS perspective, model inputs derived from multi-centre cohort study of immigrants in the UK, 20-year time horizon, costs in 2010 GB pounds. Population: immigrants arriving to UK from countries with varying TB incidence.	Two strategies: (i) NICE guidelines 2006 (ii) QFT testing for newly arrived migrants < 35 years	The two most cost-effective strategies were: Screen individuals from countries with incidence > 250/100,000: ICER of GBP 17,956 per case averted (EUR 21,736); Screen at incidence > 150/100,000: ICER of GBP 20,819 per case averted (EUR 25,202).	Moderate to large costs compared with no screening. Total costs: No screening: GBP 608,370 (EUR 736,465); IGRA (up to age 35): GBP 1,532,257 (EUR 1,854,881).
Hardy et al. 2010 [40]	Certainty of evidence: low. No allowance was made for uncertainty in the estimates of costs and consequences. No PSA was performed. Not applicable – no sensitivity analyses undertaken. No cost-effectiveness results presented.	Method: cost analysis based on a cohort study at the Leeds TB screening service for immigrants from high-incidence countries. Population: immigrants from high-incidence countries (TB incidence > 200/100,000) to Leeds, England.	Two strategies: (i) QFT first; CXR if QFT-positive (Leeds protocol) (ii) CXR first; TST if pregnant, < 16-years-old, or from sub-Saharan Africa; QFT if positive TST (NICE protocol)	Overall, the Leeds protocol was cheaper and identified more cases of LTBI (n = 105) than the NICE protocol (n = 83).	Moderate to large costs compared with no screening. Total cost of Leeds protocol in 280 patients: GBP 9,782 (EUR 12,815); Total cost of NICE protocol in 280 patients: GBP 13,347 (EUR 17,487). All individuals from countries with incidence > 200/100,000
Brassard et al. 2006 [42]	Certainty of evidence: low. Limited allowance was made for uncertainty in the estimates of costs and consequences. No PSA was performed. Limited sensitivity analyses undertaken, no justification for ranges used. Net savings were sensitive to rates of hospitalisation test performance characteristics.	Method: cost–benefit analysis of school-based screening programme, 20-year time horizon, 3% discount rate; results in Canadian dollars. Population: newly arrived immigrant children to Canada (aged 14–18 years).	Two strategies: (i) LTBI school screening (ii) Passive case finding and active TB treatment	Net savings from both school-based screening and associate investigations. Total net savings from conducting both programmes of CAD 363,923 (EUR 296,803)	Moderate to large costs: Total cost of school-based screening: CAD 126,871 (EUR 103,471); Total cost of associated investigations: CAD 66,590 (EUR 54,308).
Porco et al. 2006 [43]	Certainty of evidence: low. Allowance was made for uncertainty in the estimates of costs and consequences; ranges provided. No PSA was performed. Limited justification for ranges used in sensitivity analyses. Cost-effectiveness results were mostly robust but sensitive to changes in hospitalisation rates for actively found and passively found cases; INH hepatitis rates; proportion of active cases identified.	Method: decision-analytic model, 20-year time horizon, US domestic health payer perspective, 3% discount rate; results presented in US dollars. Population: immigrants to the US.	Two strategies: (i) Follow-up programme and LTBI treatment of contacts (ii) No follow-up of notifications	Costs per QALY range: USD 7,000 (EUR −6,761) to USD 72,000 (EUR 69,549): Population of 40% TB patients (range dependent on proportion of active cases; range 0–2%). The treatment intervention was cost-saving if the fraction of active cases was 2.5% or above.	Total costs not provided. Resource requirements unclear.
Khan et al. 2002 [44]	Certainty of evidence: moderate allowance was made for uncertainty in the estimates of costs and consequences; ranges provided. Monte Carlo simulation was performed. Justification for ranges used in sensitivity analyses was provided. Cost-effectiveness results were mostly robust, however sensitive to changes in INH or RMP resistance; cost of RMP.	Method: decision-analytic model, region-specific resistance profiles constructed from a cross-sectional dataset. Time horizon was average life expectancy of foreign-born persons in the US minus median age of migrants. 3% discount rate; results reported in US dollars. Population: newly arrived immigrants to the US.	Four strategies: (i) No intervention (ii) TST followed by treatment with INH (iii) Treatment with RMP, (iv) Treatment with RIF plus PZA for those with a positive test result	A strategy of detecting and treating LTBI among immigrants would result in both health benefits and economic savings. RIF may only be superior to INH in migrants of certain national origins; this analysis includes a comparison of INH with a hybrid RIF/PZA regime.	Costs varied considerably by country of origin and prevalence. Costs for INH treatment: South Korea: USD 6.2 million (EUR 6,517,956); Mexico: USD 60.9 million (EUR 64,023,151). Costs for RIF treatment: South Korea: USD 6.9 million (EUR 7,253,854); Mexico: USD 69.7 million (EUR 73,274,443). Note: costs varied with size of immigrant population and prevalence.
Chang et al. 2002 [45]	Certainty of evidence: low. No allowance was made for uncertainty in the estimates of costs and consequences. No PSA was performed. No sensitivity analyses undertaken. Net savings were not tested for plausible changes in costs or benefits.	Method: cost-benefit study of 706 foreign-born students in a Maryland school; results presented in US dollars. Population: foreign-born school students in the US.	Two strategies: (i) No screening (ii) TST screening	Net benefit of USD 65,733 (EUR 70,675) of the TST screening and treatment intervention.	Moderate costs. Total cost of USD 32,617 (EUR 35,069) for TST screening and follow up treatment in 706 foreign-born school students.
Shah et al. 2012 [34]	Certainty of evidence: high. Allowance was made for uncertainty in the estimates of costs and consequences. PSA was performed. Sensitivity analyses undertaken and justification for ranges of model estimates provided. Cost-effectiveness results were robust to all changes in key model parameters.	Method: decision-analytic model. CEA undertaken from a US health system perspective, over a 1- and 5-year time horizon. Costs presented in 2012 US dollars, discounted at 3% per annum. Population: individuals referred to public health clinics with suspected LTBI on the basis of a positive TST.	Two strategies: (i) Treat all TST-positive referrals (ii) Treat those with positive results on adjunctive QFT-GIT testing	USD 1,202 (EUR 983) per QALY gained with TST + QFT vs TST alone.	Negligible costs and savings. Resource use, TST alone: symptom screen, CXR, liver chemistries, + LTBI treatment. TST + QFT-GIT resource use: QFT, symptom screen, CXR, liver chemistries, + LTBI treatment only if QFT positive. Total costs per individual at 1 year USD 360 (EUR 294); per person for TST alone: USD 370 (EUR 302); per person for TST + QFT: USD 10 (EUR 8) difference.
Mancuso et al. 2011 [37]	Certainty of evidence: moderate Allowance was made for uncertainty in the estimates of costs and consequences. PSA was not performed. Justification for ranges in sensitivity analyses was provided. Cost-effectiveness results were sensitive to changes in prevalence of LTBI; test performance characteristics; cost of tests.	Method: decision-analytic Markov model. CEA undertaken from a US societal perspective, over a 20-year time horizon. Costs presented in 2009 US dollars, discounted at 3% per annum. Population: recruits entering the US military at Fort Jackson, SC, US.	Four strategies: (i) Targeted screening (ii) Universal screening with IGRA +/− TST in low prevalence US military recruits (iii) Sequential testing strategies (iv) No screening	Targeted testing the most cost-effective vs no screening: ICER: USD 285,777 (EUR 246,015)/case prevented Sequential strategies and universal QFT testing are dominated.	Large costs compared with no screening. Screening per 200,000 recruits: No screening: USD 1,540,000 (EUR 1,325,731); Targeted screening: USD 6,580,000 (EUR 5,664,487); Targeted TST + QFT: USD 13,620,000 (EUR 11,724,972); Targeted TST + T-SPOT: USD 13,760 (EUR 11,845); Universal TST: USD 14,720 (EUR 12,671).
Deuffic-Burban et al. 2010 [39]	Certainty of evidence: moderate. Allowance was made for uncertainty in the estimates of costs and consequences. PSA was not performed. Justification for ranges in sensitivity analyses was provided. Cost-effectiveness results were sensitive to changes in TST specificity; costs of treatment.	Method: decision-analytic Markov model. CEA undertaken from a French healthcare payer's perspective, over a patient's lifetime, ca 48 years time horizon. Costs presented in 2007 Euros, discounted at 3% per annum. Population: adults in close contacts with BCG vaccinated.	Four strategies: (i) No testing (ii) TST (iii) TST + QFT for close contacts who have been BCG vaccinated (iv) QFT	TST had higher costs and lower efficacy than QFT (i.e. dominated). TST + QFT: ICER of EUR 560 (EUR 581^a^) /YLG compared with no testing; QFT = ICER of EUR 730 (EUR 757ꝉ) YLG compared with TST + QFT.	Negligible costs and savings. The discounted direct medical lifetime costs of care per patient were: No testing EUR 417 (EUR 432^a^); TST EUR 476 (EUR 493^a^); QFT EUR 443 (EUR 459^a^); TST + QFT EUR 435 (EUR 451^a^).
Pooran et al. 2010 [38]	Certainty of evidence: moderate. Allowance was made for uncertainty in the estimates of costs and consequences. PSA was not performed. Justification for ranges in sensitivity analyses was provided. Cost-effectiveness results were sensitive to changes in LTBI prevalence; test sensitivity and specificity; LTBI treatment costs.	Method: decision analytic model. CEA undertaken from a UK healthcare perspective, over a 2-year time horizon. Costs presented in 2008 GB pounds, no discounting. Population: close contacts of individuals with TB in the UK.	Five strategies: (i) TST alone (ii) T-SPOT.TB assay alone (iii) TST followed by T-SPOT.TB assay when TST was positive (TST/T-SPOT.TB) (iv) Quantiferon-TB-Gold-In-Tube (QFT-GIT) alone (v) TST followed by QFT-GIT when TST was positive	Incremental cost per active case prevented (compared with no screening): TST: GBP 47,840 (EUR 60,938); QFT-GIT: GBP 42,051(EUR 53,564); T-SPOT.TB: GBP 39,712 (EUR 50,584); TST/QFT-GIT: GBP 37,699 (EUR 48,020); TST/T-SPOT.TB: GBP 37,206 (EUR 47,392). In most cases T-SPOT.TB dual screening was the most cost-effective strategy, TST alone the least cost-effective.	Large costs compared with no screening. Total costs including treatment, follow-up and test costs per 1,000 contacts: T-SPOT.TB: GBP 203,983 (EUR 259,832); QFT-GIT: GBP 202,921 (EUR 258,479); TST: GBP 199,589 (EUR 254,235); TST/T-SPOT.TB: GBP 162,387 (EUR 206,847); TST/QFT-GIT: GBP 157,048 (EUR 200,047); No screening: GBP 57,148 (EUR 72,794).

BCG: Bacillus Calmette–Guérin; CAD: Canadian dollar; CEA: cost effective analysis; CXR: chest radiography; ELISpot: Enzyme-Linked ImmunoSpot; GBP: British pound; EUR: Euro; HIV: human immunodeficiency virus; ICER: incremental cost-effectiveness ratio; IGRA: Interferon Gamma Release Assay; INB: incremental net benefit; INH: isoniazid; LTBI: latent tuberculosis infection; NHS: National Health Service; NICE: The National Institute for Health and Care Excellence; PSA: probabilistic sensitivity analysis; PZA: pyrazinamide; QALY: quality-adjusted life years; Gold In-Tube; QFT: QuantiFERON; QFT-GIT: QuantiFERON-TB, Gold In-Tube; RIF: rifampicin; RMP: rifampicin; SC; South Carolina; TB: tuberculosis; TST: tuberculin skin test; T-SPOT.TB: ELISPOT assay for tuberculosis; UK: United Kingdom; US: United States; USD: US dollar; YLG: years of life gained.

The Drummond Criteria include [26]: (i) Was a well-defined question posed in answerable form? (ii) Was a comprehensive description of the competing alternatives given (i.e. can you tell who did what to whom, where, and how often)? (iii) Was the effectiveness of the programme or services established? (iv) Were all the important and relevant costs and consequences for each alternative identified? (v) Were costs and consequences measured accurately in appropriate physical units (e.g. hours of nursing time, number of physician visits, lost working days, gained life years)? (vi) Were the cost and consequences valued credibly? (vii) Were costs and consequences adjusted for differential timing? (viii) Was an incremental analysis of costs and consequences of alternatives performed? (ix) Was allowance made for uncertainty in the estimates of costs and consequences? (x) Did the presentation and discussion of study results include all issues of concern to users?

All currencies were converted to 2015 Euros using the Cochrane web-based currency conversion tool: https://eppi.ioe.ac.uk/costconversion/default.aspx. Resource use was expressed in cost per person and classified as low (savings or ≤ USD 1,000/person or EUR 808), moderate (USD 1,000–100,000/person or EUR 808–80,845) or high (USD ≥ 100,000/person or EUR > 80,845).

^a^ 2007 Euros were converted to 2015 Euros for comparability.

Four studies reported that screening with a single-step IGRA was less costly or more cost-effective relative to TST screening in migrants to prevent incident TB [33,35,36,48]. In one study in the US by Linas et al., a single IGRA dominated TST in all comparisons. However, IGRA was only cost-effective at a willingness-to-pay threshold of less than USD 75,000 per QALY (EUR 62,496/QALY) compared with no screening among migrants younger than 25 years of age, with an incremental cost-effectiveness ratio (ICER) ranging from USD 52,900–74,800 per QALY (EUR 44,080–62,329/QALY). For migrants older than 45 years, the intervention was unlikely to be cost-effective, with an ICER for IGRA vs no screening between USD 103,000–283,000 per QALY gained (EUR 85,827–235,817/QALY) [36]. Two studies conducted in the United Kingdom (UK) by Pareek et al. found that performing an IGRA in migrants aged 16–35 years and originating from countries with a TB incidence of > 150 per 100,000 was the most cost-effective LTBI strategy, with an ICER of ca GBP 20,000 (EUR 24,211) to GBP 30,000 (EUR 36,317) per active TB case prevented [35,48].

Other studies investigated the optimal LTBI testing strategy in different high-risk populations such as contacts of active cases or migrants from TB-endemic countries [38,39,41]. Sequential TST/IGRA testing was preferred over single TST or IGRA, especially in those who had a high likelihood of a true positive TST (LTBI prevalence > 5%) and were BCG-vaccinated after infancy [39,41]. Oxlade et al. found that sequential TST-IGRA screening was cost-effective compared with single-step IGRA screening. That study suggested that it was most cost-effective to use an IGRA to screen TST-positive cases, and that IGRA screening was favoured only among those who had received BCG vaccination after infancy [41]. In a French study by Deuffic-Burban, sequential TST-IGRA screening was a more cost-effective strategy for BCG-vaccinated close contacts of active TB patients than IGRA alone [39]. For TST-IGRA compared with no testing, the ICER was EUR 560 (EUR 581, as per 2015) per year of life gained (YLG), and for IGRA compared with TST-IGRA, the ICER was EUR 730 (EUR 757) per YLG in the scenario when LTBI prevalence was more than 5%. This was robust across a wide range of LTBI prevalence. In the study by Pooran et al., sequential TST-IGRA testing was more cost-effective compared with no screening or single-step TST, with an incremental cost per active case prevented of GBP 37,699 (EUR 48,020) to GBP 37,206 (EUR 47,392) among contacts of active TB [38].

## Discussion

There were no single studies that directly addressed the effectiveness of latent TB screening programmes on the health outcomes of migrants. Therefore, we evaluated the LTBI screening chain of evidence. The majority of TB cases in low TB incidence countries in the EU/EEA occur in migrants born in countries with higher TB incidence and occur primarily due to reactivation of latent infection. The tools to detect and treat LTBI, however, have many limitations. IGRA and TST have high sensitivity to detect LTBI but they both predicted the development of active TB poorly [20,27,28]. All latent TB therapies were equivalent but their effectiveness in preventing the development of active TB was only low to moderate [29,30]. RIF regimens may be preferable because they have considerably lower hepatotoxicity and higher treatment completion rates than INH [30]. The LTBI care cascade is weak as only a minority of patients (both general population and migrants) eligible for LTBI screening actually complete LTBI treatment [31]. Limited economic analyses of LTBI screening among migrants suggest that targeted screening for young migrants from high TB incidence countries (> 150/100,000) is the most cost-effective strategy [35].

The WHO *End TB Strategy*, with a goal to eliminate TB by 2050, highlights the need to decrease the substantial reservoir of individuals with latent TB infection at risk of progression to active TB [49,50]. A substantial proportion of migrants were born in high TB burden countries and many have latent TB infection (26–46%) [4,13]. A major challenge is identifying those at highest risk for progression to active disease so that targeted programmes can be developed that will promote the health of migrants and have the highest public health impact.

Ca 5–15% of individuals with latent infection will develop active TB during their lifetime [51,52]. The groups at highest risk of progression to active TB disease are those with immunosuppressive conditions (i.e. HIV infection, immunosuppressive therapies with anti-tumour necrosis factor treatment, organ transplantation or dialysis) and those infected recently [6]. The risk of disease progression is greatest close to the time of infection, with almost half of disease progression cases occurring within the first 2–3 years after exposure [53]. Migrants arriving from endemic areas have the highest rates of active TB soon after arrival in host countries, which is probably due to recent exposure in their countries of origin. Fifty per cent of cases, however, occur 5 or more years after arrival and the risk remains elevated throughout their lifetime [54-57]. Being an asylum seeker or refugee, TB exposure during crowded conditions or perilous journeys to host countries, or recent travel back to TB-endemic countries of origin may also increase the risk of active TB in the migrant population [58-60]. The complex epidemiology of TB among migrants needs to be taken into consideration when developing LTBI programmes for this population to ensure the highest individual and public health benefit. The lack of robust population-based data is, however, a major obstacle in developing targeted LTBI programmes for migrants. Estimates on the individual, combined and attributable population contribution of each of these risk factors to developing TB among migrants will be required. There are also few studies on cost-effectiveness to inform latent TB programmes concerning migrants. Only two studies conducted in the UK specifically addressed which migrant groups should be targeted for LTBI screening and treatment [35,48]. These results however, may not be generalisable to all EU/EEA countries as willingness to pay thresholds, per capita health care expenditures, and health priorities vary between countries.

In addition to these data gaps, the tools to diagnose and treat latent TB have limitations. The LTBI care cascade is weak, lowering the effectiveness and impact of screening programmes. Both TST and IGRA poorly predict the small proportion (< 15%) of those infected with TB who will progress to active disease. As a consequence, a large number of people need to be screened and treated to prevent one case of active TB [6]. Operational issues related to TST and IGRA may decrease screening uptake: The TST requires a second visit 48–72 h after the first visit to read the skin test induration (test result) and IGRA testing is generally costlier than TST and may not be as widely available in EU/EEA countries [61]. Patients with latent TB are asymptomatic and thus long treatment regimens ranging from 3 to 9 months lead to poor treatment completion [32]. The latent TB care cascade involves several steps including identifying patients in need of screening, offering screening and treatment by providers, and uptake and completion of screening and treatment by patients. This process requires the understanding and engagement of patients and providers. The low proportion of those eligible for screening who complete LTBI treatment is a result of losses at every point of the care cascade because of barriers at patient, provider and structural level [31].

Migrants encounter several barriers in accessing healthcare and consequently, treatment initiation (23–97%) and completion rates (7–86%) are variable [21,32,62,63]. In addition, practitioners may lack adequate knowledge of which migrants should be screened and treated [21,64]. Addressing barriers at both the patient and provider level will therefore be required to strengthen the LTBI care cascade and to ensure individual and public health benefits of LTBI programmes. With the adoption of the WHO *End TB Strategy* there is recognition of the importance of scaling up preventive therapy. Less than half of EU/EEA countries, however, have LTBI programmes for migrants and there are numerous challenges to developing and implementing new programmes [11,14,15]. These include the heterogeneity of populations and migrant subgroups affected by TB in individual EU/EAA countries as well as economic and operational considerations. LTBI screening programmes will therefore need to be tailored to the local TB epidemiology in host countries, the TB risk in migrant sub-groups, and implementation based on the health priorities and economic and healthcare capacity in each setting [2,3].

### Study limitations

Our study was limited by the fact that we did not retrieve any studies that directly estimated the effectiveness of LTBI screening programmes among migrants or the general population. There are limited data on the cost-effectiveness of LTBI screening in these populations. The search was limited by the fact that it was conducted only up until May 2016 and that we only included studies published in English or French. A recent narrative review of the effectiveness and cost-effectiveness, however, found similar literature and findings as our study [65]. Our findings are further limited by the low or very low quality of most of the original studies that were included in the systematic reviews.

### Evidence gaps and future directions

Better evidence is urgently needed on the individual, combined and attributable population contribution of risk factors leading to progression from LTBI to active TB in migrants. Intervention studies that determine how to improve the identification of target populations and retain them in care along with cost-effectiveness studies that use this intervention and the epidemiological data will be needed to develop programmes with the highest impact. Ultimately, better diagnostic tests that accurately predict those individuals who will develop active TB as well as shorter, well-tolerated and more effective treatment to promote adherence, will be needed to achieve TB elimination.

## Conclusions

The latent TB burden among migrants needs to be addressed in order to promote the health of this population and to achieve TB elimination in the EU/EEA. At present, broad implementation of LTBI screening and treatment programmes is hindered by the large pool of migrants with LTBI (a small proportion of whom will develop active TB), diagnostic tests that poorly predict which individuals will develop active TB, long LTBI treatment regimens, as well as several patient, provider and institutional barriers that lead to poor uptake of screening and treatment completion. Despite these limitations, migrant-focused latent TB screening programmes may be effective and cost-effective if they are highly targeted and well implemented.
